# Perovskite Nanocrystal Self-Assemblies in 3D Hollow Templates

**DOI:** 10.1021/acsnano.4c07819

**Published:** 2025-01-13

**Authors:** Etsuki Kobiyama, Darius Urbonas, Benjamin Aymoz, Maryna I. Bodnarchuk, Gabriele Rainò, Antonis Olziersky, Daniele Caimi, Marilyne Sousa, Rainer F. Mahrt, Maksym V. Kovalenko, Thilo Stöferle

**Affiliations:** † IBM Research Europe—Zurich, Säumerstrasse 4, 8803 Rüschlikon, Switzerland; ‡ Institute of Inorganic Chemistry, Department of Chemistry and Applied Bioscience, ETH Zurich, 8093 Zurich, Switzerland; § Laboratory of Thin Films and Photovoltaics, Empa—Swiss Federal Laboratories for Materials Science and Technology, 8600 Dübendorf, Switzerland

**Keywords:** colloidal nanocrystals, nanocrystal assembly, nanocrystal superlattice, templated assembly, lead halide perovskites, superfluorescence

## Abstract

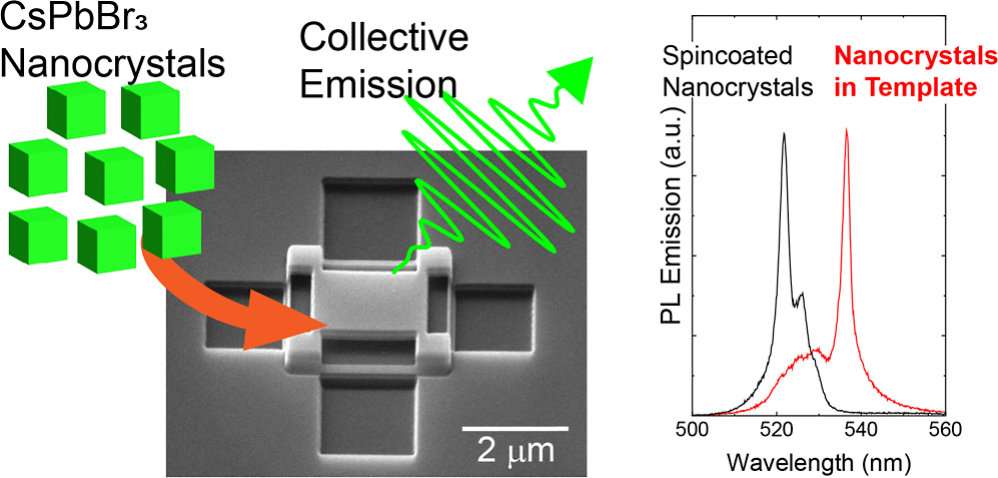

Highly ordered nanocrystal (NC) assemblies, namely, superlattices (SLs), have been investigated as materials for optical and optoelectronic devices due to their unique properties based on interactions among neighboring NCs. In particular, lead halide perovskite NC SLs have attracted significant attention owing to their extraordinary optical characteristics of individual NCs and collective emission processes like superfluorescence (SF). So far, the primary method for preparing perovskite NC SLs has been the drying-mediated self-assembly method, in which the colloidal NCs spontaneously assemble into SLs during solvent evaporation. However, this method lacks controllability because NCs form random-sized SLs at random positions on the substrate, rendering NC assemblies in conjunction with device structures, such as photonic waveguides or microcavities, challenging. Here, we demonstrate template-assisted self-assembly to deterministically place perovskite NC SLs and control their geometrical properties. A solution of CsPbBr_3_ NCs is drop-casted on a substrate with lithographically defined hollow structures. After solvent evaporation and removal of excess NCs from the substrate surface, NCs remain only in the templates, thereby defining the position and size of these NC assemblies. We performed photoluminescence (PL) measurements on these NC assemblies and observed signatures of SF, similar to those in spontaneously assembled SLs. Our findings are crucial for optical devices that harness embedded perovskite NC assemblies and enable fundamental studies on how these collective effects can be tailored through the SL geometry.

Colloidal semiconductor nanocrystals (NCs) are often referred to as “artificial atoms” or “quantum dots” since their physical properties originate from quantized electronic states.^1,2^ Because of the outstanding optical properties and broad range of potential applications, extensive research has been carried out. Thanks to the recent advances in synthesis technology, NC ensembles with very narrow size distribution can be produced by easy and cost-effective methods,^3^ allowing to tailor meso-structured materials by using individual NCs as building blocks. One of the best known meso-structured materials made out of colloidal NCs are highly ordered NC solids, so-called NC superlattices (SLs).^4,5^ SLs exhibit not only physical properties arising from individual NCs but also unique properties based on inter-NC interactions in analogy to characteristic properties of bulk solids, which arise from interatomic interactions.^6−8^ Because it is possible to engineer the optical and electronic properties of SLs by altering their composition and/or orientation,^9−11^ NC SLs have been investigated to create materials with tailored physical properties. One of the most striking impacts of SLs on the optical properties is the emergence of cooperative photon emission resulting from enhanced inter-NC interactions. Due to the closely packed arrangement of NCs,^5,12^ an excited electric dipole in a NC can coherently interact with dipoles excited in neighboring NCs. One typical cooperative photon emission process, namely, superfluorescence (SF),^13,14^ has been observed from SLs consisting of lead halide perovskite NCs.^15−17^ Due to its characteristic fast and intense emission with narrow line width, SF has been of interest for ultrafast photonic technology as well as for novel bright (quantum) light sources. Thus far, the most used method for SL fabrication has been the drying-mediated self-assembly method.^5,18^ In this process, NCs spontaneously arrange into highly ordered structures while the solvent slowly evaporates.^19^ However, because the self-assembly process involves complex interactions among multiple different types of driving forces,^5,18^ it has been challenging to deterministically position and shape SLs in order to integrate them into micro- or nanodevice structures such as photonic waveguides or resonators. For unordered colloidal NC assemblies, different template-assisted deposition techniques have been developed in recent decades. These ranged from the deposition of individual NC in arrays^20,21^ through convective, capillary assembly over deterministic, number- and geometry-controlled assemblies^22−24^ to large clusters in photonically functional nanostructures.^25−27^ Nevertheless, a controlled template-assisted assembly of SLs exhibiting cooperative optical emission is missing. Here, we introduce hollow templates with three-dimensional confinement and demonstrate template-assisted self-assembly to achieve precise positioning and size definition of perovskite NC SLs. We study the effect of different solvents, NC ligand molecules, and template geometries on the assembly process and yield. Additionally, using time-resolved spectroscopy at cryogenic temperatures, we optically characterize the SLs and observe signatures of SF that are consistent with results from spontaneously assembled ordered SLs^15,16^ but clearly distinct from spin-coated NC films.

## Results and Discussion

**Figure 1:**
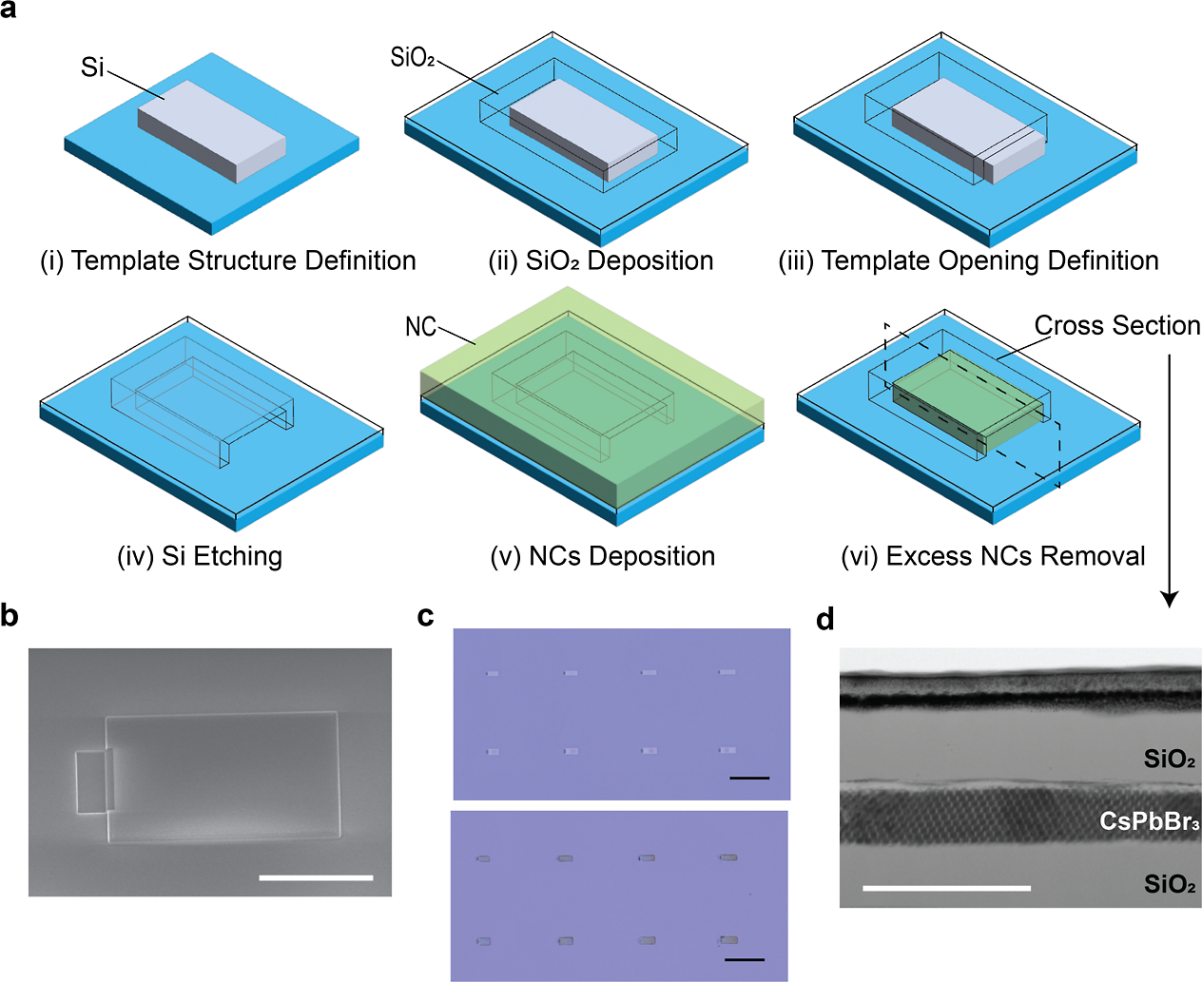
(a) Schematic illustration of the process flow to fabricate transparent, hollow templates that are subsequently filled with NCs. (b) SEM image of a template structure. Scale bar: 5 μm. (c) Optical images of an array of template structures before NCs deposition (Top) and template-assisted NC assemblies after NCs deposition (Bottom). Scale bar: 25 μm. (d) Bright-field STEM image showing a cross section of a template-assisted NC assembly. Scale bar: 200 nm.

For the template structure fabrication, we adapted the processes developed for template-assisted selective epitaxial growth of III–V semiconductors on silicon.^28^ The fabrication steps of the template structures are sketched in Figure 1a. (i) Silicon-on-insulator (SOI) substrates or glass substrates with a Si layer on top were patterned by using e-beam lithography (EBL) and reactive ion etching (RIE). The Si layer has a thickness of 220 nm, defining the height of the hollow space within the template structures. (ii) To realize the walls and ceiling of the templates, conformal atomic layer deposition (ALD) and electron-beam evaporation were used to deposit a 200 nm thick SiO_2_ layer over the structures. (iii) Template openings were defined by EBL and RIE of the encapsulating SiO_2_ layer. (iv) The exposed Si layer was dry-etched with XeF_2_ gas. A scanning electron microscopy (SEM) image of the final template structure is shown in Figure 1b. (v) After the templates were constructed, a CsPbBr_3_ NC solution in toluene was drop-cast on the substrates, and the solvent was slowly evaporated in the toluene atmosphere. (vi) Finally, excess NCs on the substrate surface are removed by applying an optics cleaning polymer (Red First Contact Polymer, Photonic Cleaning Technologies).

The CsPbBr_3_ NCs with a monodisperse size distribution (8.7 ± 0.6 nm) were produced leveraging the recently reported room-temperature synthesis platform based on PbBr_2_/trioctylphosphine oxide (TOPO) molecular adducts as the precursor, wherein the formation of NCs is precisely adjustable owing to slower reaction kinetics.^29^ The ligands used in the TOPO/PbBr_2_ method are loosely bound TOPO and the dialkylphosphinate anion and, hence, are readily displaced with a ligand of choice. In this study, didodecyldimethylammonium bromide (DDAB), oleic acid and oleylamine (OA/OLA) with DDAB, or phosphatidylserine (Ptd-l-Ser) were added at room temperature at the end of the NC formation, before purification and isolation of NCs (see Methods and Figure S1). The resulting NC shape was governed by the added ligand: Ptd-l-Ser acts as lecithin,^29^ yielding truncated (pseudospherical) NCs. On the contrary, primary and tertiary ammonium ligands induce NC refaceting into a sharp cuboid morphology as these ligands stabilize (100) pseudocubic surface facets of orthorhombic CsPbBr_3_.

Inspection of the SL formation has been first conducted by an optical microscope. Bright-field optical images show the hollow template structures in white color before the NC deposition, while the substrate is shown as light blue color (Figure 1c Top). After the NC drop-casting and the excess NCs removal processes, the templates are displayed in green because they are partially or fully filled with green perovskite NCs (Figure 1c Bottom). Furthermore, we used a scanning transmission electron microscope (STEM) to examine the cross section of the template-assisted NC assemblies (see Methods). Within the templates, the NCs form ordered structures, as shown in Figures 1d and S2. The NC layer is 60–70 nm thick, indicating an air gap between the perovskite NC layer and the upper window and an incomplete filling of the templates by the NCs. It is worth noting that because the sidewalls of the templates were removed and the top window dropped down after the STEM lamella preparation, the air gap is not visible in the STEM image.

In addition, we conducted grazing-incidence small-angle X-ray scattering (GISAXS) measurements to investigate the long-range ordering of template-assisted NC assemblies (Figure S3a). The GISAXS signal, which averages over hundreds of template structures, exhibits three scattering rings corresponding to a randomly oriented cubic SL domain. These three rings originate from the reflections indexed as (100), (200), and (300), with a lattice constant of 10.6 nm. This lattice constant is attributed to the size of the NCs with their ligand shells (∼8.7 + 2 × 1.0 nm), acting as the unit cell of the SL. The scattering rings indicate that there is no global orientational alignment of the SLs as they form independently. The lower bound on the number of SL domains contributing to the pattern corresponds to the number of templates within the X-ray spot size of 2.0 × 0.3 mm, which is up to 200 templates. In contrast, no specific scattering pattern is observed for an empty template chip without NC incorporation (Figure S3b), confirming that it is NC assemblies and not background artifacts that lead to the scattering pattern in Figure S3a.

We also investigated a spin-coated NC film with GISAXS (Figure S3c). Due to the much larger amount of NCs compared to that of the templates, the signal is overall much stronger. The measurement exhibits intense scattering rings and spots that indicate long-range order with the same lattice constant, confirming the strong tendency of the NCs to self-assemble into ordered, large thin-film SL domains during drying. However, for the spin-coated films (Figure S4), we did not observe micron-sized SL domains such as the ones shown in Figure S5a by optical microscopy inspection. Hence, we interpret the results that SL domains can be formed within spin-coated films due to the cubic shape of NCs, but domain sizes and/or the ordering in the third dimension presumably remain too small to support collective emission processes such as SF, as discussed below with the optical spectroscopy results.

### Influence of Solvent and Ligands

**Figure 2:**
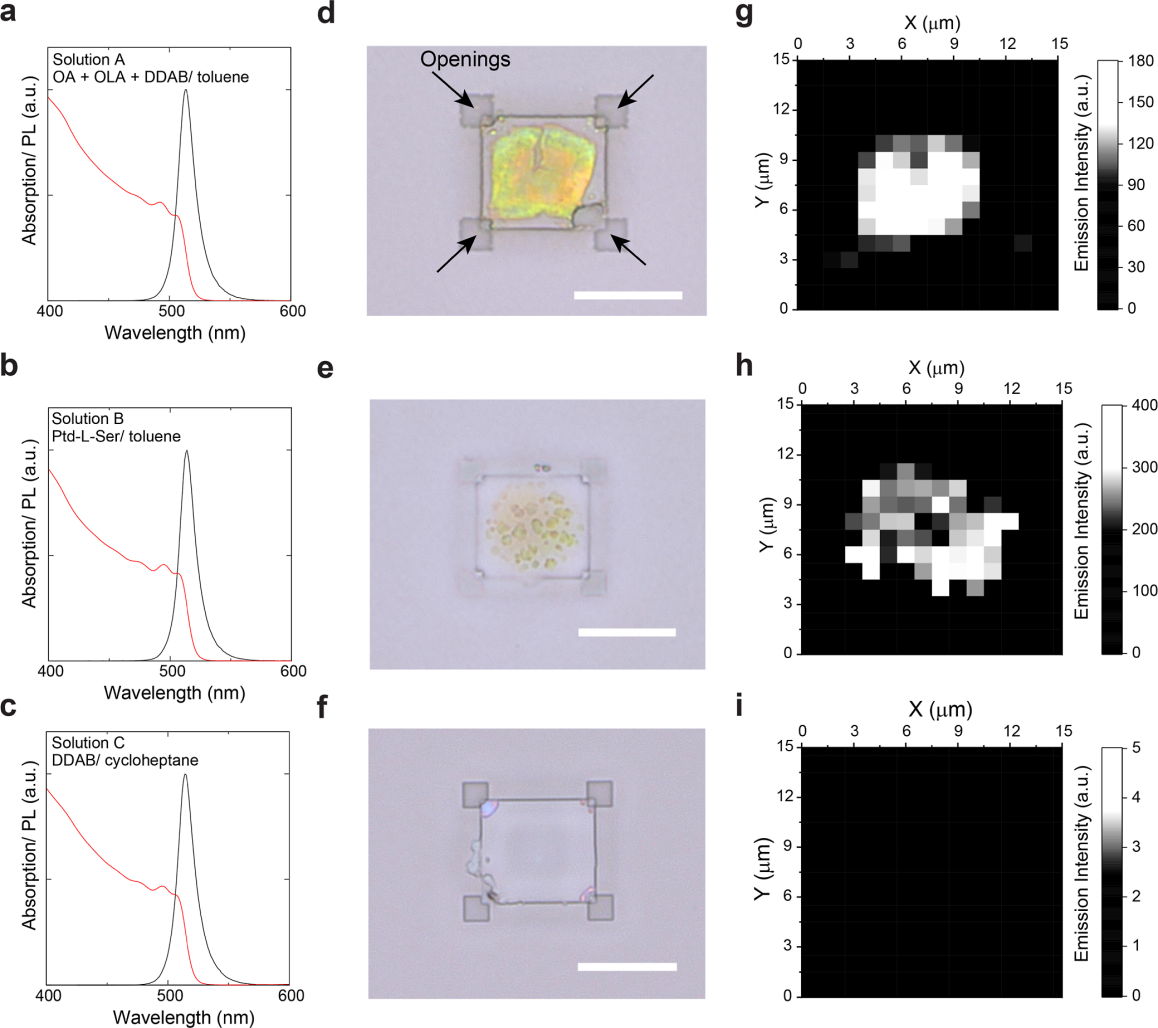
Absorption (red solid lines) and PL (black solid lines) spectra of NC solutions (a–c), optical microscopic images (d–f, scale bars: 10 μm), and spatially resolved PL maps (g–i) of NC assemblies prepared by the template-assisted method. The surface capping ligands and solvent of the solution are (a,d,g) ligands: oleic acid (OA) + oleylamine (OLA) + didodecyldimethylammonium bromide (DDAB), solvent: toluene, (b,e,h) ligands: phosphatidylserine (Ptd-l-Ser), solvent: toluene, and (c,f,i) ligands: DDAB, solvent: cycloheptane.

Since the self-assembly process is driven by interactions at the interfaces of vapor–liquid, ligand–ligand, ligand–solvent, solvent–substrate/template, and ligand–substrate/template, the surface characteristics of NCs, solvent, substrates, and templates play important roles. However, the complexity of the liquid with its manifold of parameters (many of which are not known precisely) and its interplay with nanoscale confinement geometry renders accurate modeling difficult.^30^ Therefore, we investigate empirically the influence of the main factors. First, we studied combinations of NC surface capping ligands and the solvent. Figure 2a–c shows the absorption and PL spectra of different NC solutions. The solution in Figure 2a is composed of a mixture of OA, OLA, and DDAB as capping ligands and toluene as the solvent (denoted as solution A). The solution in Figure 2b consists of Ptd-l-Ser as the capping ligand and toluene as the solvent (denoted as solution B). The solution C in Figure 2c is composed of DDAB as the capping ligand and cycloheptane as the solvent. These three different types of solutions all exhibit the PL emission peak at 514 nm with a fwhm of 15.8 ± 0.8 nm and an absorption band-edge at 513.8 ± 0.8 nm. Based on the similarity of the spectral features of the three solutions, we confirmed that the size distributions of the three different NC ensembles are the same, and the different ligands/solvents do not affect the absorption and PL spectra of the NCs.

We chose these solvent/ligand combinations to exemplarily investigate how differences in wettability and ligand interactions affect the assembly results. DDAB or the combination of OA, OLA, and DDAB was chosen because NCs covered by these ligands form in a nontruncated cuboid shape, which is necessary for obtaining the desired SL structure.^16^ On the other hand, NCs capped with Ptd-l-Ser usually do not assemble with the standard drying-mediated method. However, we nevertheless chose Ptd-l-Ser to study if the template structures, due to their strong 3D confinement, could foster the NC assembly process compared to the nonconfined process. When drop-casting on TEM grids (Figure S1d–f), different assembly morphologies become evident.

Figure 2d–f shows the optical images of typical template-assisted NC assemblies fabricated from the abovementioned three different solutions. With solution A, the NCs formed a single assembly in the template structure and filled the center part of the template structure (Figure 2d), while the NCs assembled into multiple (sub)micrometer-sized clusters in the case of solution B (Figure 2e). The spatial (in)homogeneity in both cases is clearly visible in the spatially resolved PL (Figure 2g,h) map as well. The NC assembly from solution A shows a homogeneous PL intensity over the assembly (Figure 2g). On the contrary, the NC assembly from solution B shows a very inhomogeneous image with several bright spots that correspond to small clusters (Figure 2h), which appear a bit merged compared to the room-temperature microscopy image due to the lower resolution of the micro-PL setup. Since the capping ligands (A: DDAB with OA and OLA, B: Ptd-l-Ser) are the only difference between solutions A and B, it is evident that the capping ligands play a significant role in the template-assisted self-assembly process. DDAB is preferred for producing homogeneous NC assemblies, whereas NCs with Ptd-l-Ser ligands did not form homogeneous NC assemblies. Presumably, that is because the polydispersity of ligands due to the variable fatty acid components in Ptd-l-Ser molecules effectively increases the NC–NC repulsion, and therefore, no NC assembly was formed.^31^

Figure 2f,i, displays the optical image and the results of the PL mapping measurements of a template device after the assembling process with solution C, respectively. The optical image does not show any greenish part, and the PL map does not show any PL signals. These results indicate that NCs are not present in the template structure. We attribute the lack of NCs to cycloheptane’s low wettability to the hydrophilic SiO_2_ surfaces due to its lower polarity than toluene, given the solvent difference between solutions A and C. Therefore, it is likely that the solution did not enter the hollow, submicrometer-high template structures. Considering the above observations, we concluded that DDAB with OA and OLA and toluene is the preferable combination of capping ligands and solvent for self-assembly in templates made from SiO_2_.

Besides, we confirmed that we can obtain similar results for both drying-mediated assembly and template-assisted assembly with two times diluted solution A (Figure S5). Therefore, even though the NC concentrations of the tested NC solutions are different by a factor up to 2.5, the difference of ligands or solvents has more significant effects on the assembling process compared to the difference in the NC concentration.

### Optimizing the Template Geometry

**Figure 3:**
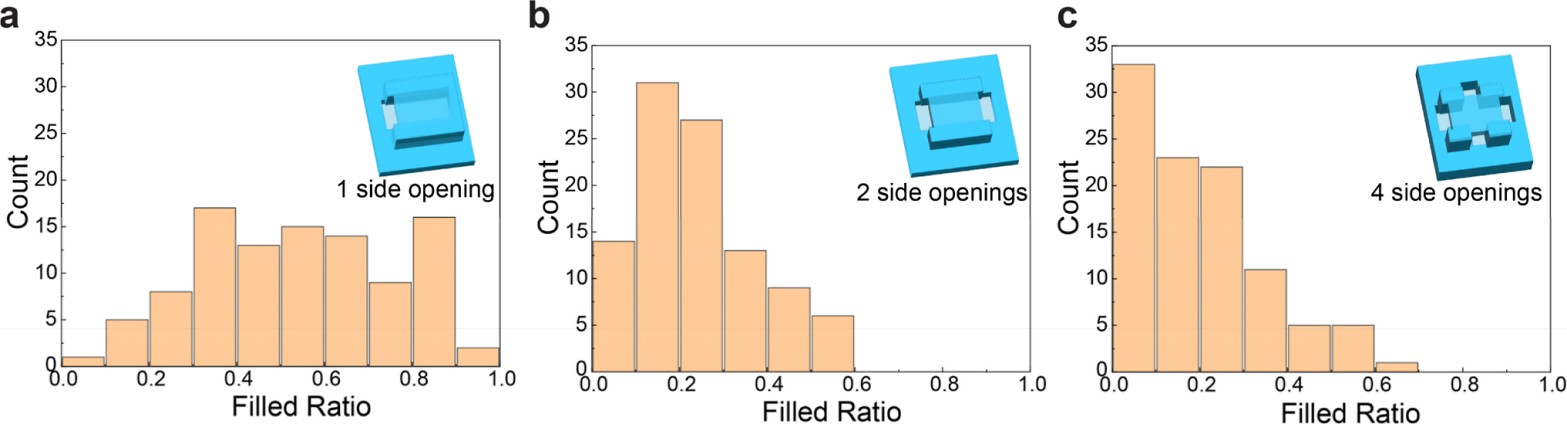
Statistical assembly yield determined from optical microscopic image analysis for different numbers of openings in the hollow template. (a–c) Histograms of the ratio of the area filled with NCs to the total area of the template structure for different numbers of openings: (a) on one side, (b) on two sides, and (c) on four sides. Insets show schematics of each template design.

Here, we studied the effects of the template geometry on the assembly process of solution A. We evaluated the yield of the process by optical image analysis (see Methods). The masked images (Figure S6) display a white color to indicate the area filled with NCs, which was identified by analyzing the RGB values of individual pixels. From the image analysis, we obtained histograms of the filling ratio (Figure 3). To determine the ideal template design, the number of opening slits through which the NC solution is inserted into the template structures was varied, as each plot’s inset illustrates. Each histogram shows the results of 100 template structures having rectangular shapes ranging in size from 1 to 100 μm^2^ and aspect ratios from 0.1 to 10 (Figure S7a). The filled ratio is defined as
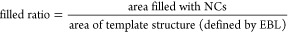


When the filled ratio equals 1, the template structure is perfectly filled by NCs, while for a filled ratio smaller than 1, the template structure is partially filled.

In the case of templates with one opening (Figure 3a), the mean value of the filled ratio is 0.54. On the other hand, the template structures with two openings (Figure 3b) and four openings (Figure 3c) result in the average filled ratio of 0.24 and 0.20, respectively. Besides, half of the structures have a filled ratio between 0.36 and 0.72 for one opening structure, while the corresponding values are 0.15–0.32 and 0.06–0.28 for two openings and four openings, respectively (Figure S7b). Based on the statistical analysis above, we conclude that one-opening template structures are the preferable design. Furthermore, we discover that elongated structures with a high aspect ratio are advantageous within the one-opening templates (Figure S7f). These findings suggest that, in contrast to the other designs, a well-defined evaporation and deposition front can propagate during the assembly process through the elongated one-opening templates. It is noteworthy that the reduced physical robustness of the structures due to the smaller sidewall areas compared to one-opening templates, which occasionally cannot withstand the cleaning process with an optics cleaning polymer, is another factor contributing to the low yields for two- or four-opening templates [step (vi) in Figure 1a].

### Superfluorescent Emission

**Figure 4:**
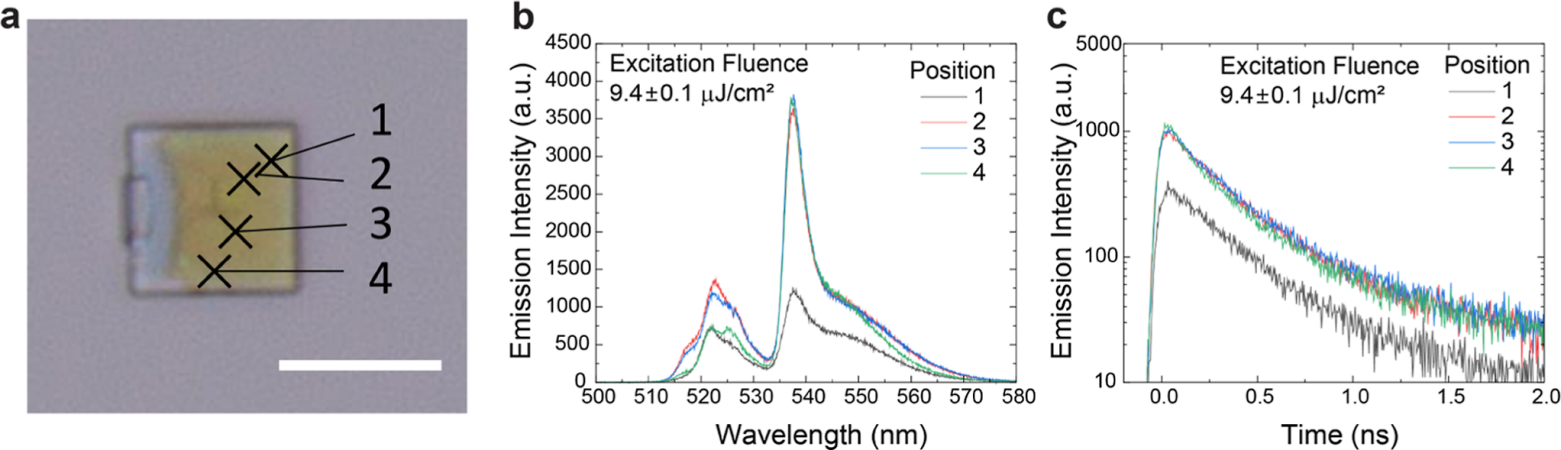
Spatially resolved PL from a NC assembly. (a) Optical microscopic image of the measured template assembly. Crosses indicate the measured positions (scale bar: 10 μm). (b) PL spectra and (c) time traces obtained at 6 K from the four different positions in the assembly. The time traces were obtained by integrating the wavelength range from 510 to 580 nm.

**Figure 5:**
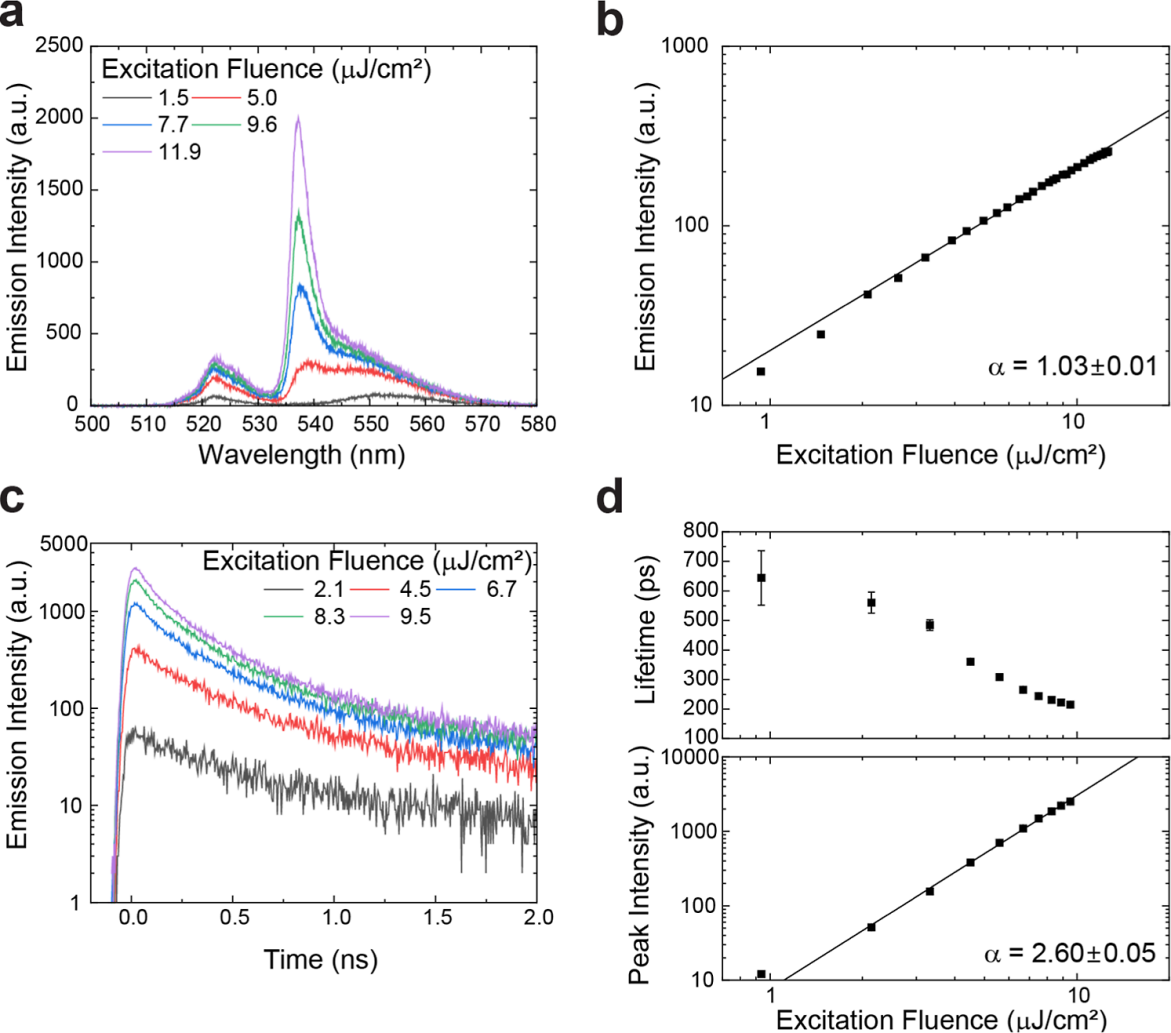
Excitation fluence dependence of PL from a NC assembly at 6 K. (a) PL spectra and (b) integrated emission intensity as a function of excitation fluence. α indicates the exponent value obtained from a power-law fit (solid line). The integrated wavelength range is from 535 to 570 nm. (c) PL time traces for different excitation fluences. The PL signal within the wavelength range from 532 to 548 nm was selected with a band-pass filter. (d) Emission lifetime (top panel) and emission peak intensity (bottom panel) as functions of excitation fluence. α indicates the exponent value obtained from a power-law fit (solid line).

Next, we investigated the optical properties of the obtained assemblies. We performed the optical characterization of template-assisted NC assemblies at 6 K because, at room temperature, the emission features are typically spectrally too broad to accurately evaluate the spatial homogeneity or spectral shifts from aggregates and because cooperative, superfluorescent emission only happens at lower temperatures (see Methods). First, we measured the emission from different positions within a single template structure to check the homogeneity of the NC assembly (Figure 4a). We chose a typically filled template with one opening and dimensions of 10 μm × 10 μm, with the excitation spot size being 4.4 μm in diameter (FWe^–2^ M). As shown in Figure 4b, the spectral features are similar at all measured positions, consisting of two-peak structures with peak center wavelengths of 522–523 and 537–538 nm. The two-peak structure is consistent with the previously reported emission spectra of superfluorescent NC assemblies, where the high-energy peak is assigned to the PL signal from an ensemble of uncoupled NCs, while the low-energy peak is assigned to the coupled NC state.^15,32^ The pronounced red tail below the band gap between 540 and 560 nm might be due to defect states occurring after the assembly. This interpretation is corroborated by the saturation of their emission (when all traps are filled) observed under higher excitation fluence (Figure 5a), while the excitonic peak around 535–540 nm emerges and grows. Additionally, we noticed that there are no appreciable variations in emission dynamics across the measured positions based on time-resolved photoluminescence (PL) measurements (Figure 4c). In the time range 0–1.0 ns, the PL time traces exhibit a single exponential decay with a lifetime of 300 ± 50 ps, consistent with the low-temperature PL decay of these CsPbBr_3_ NCs in the low-intensity, non-SF regime. The longer tail may be related to trapping or defect states-mediated delay fluorescence.^33,34^

For increasingly stronger excitation pulses, we observed significant changes in emission spectra and dynamics. Figure 5a shows the emission spectra from a single measurement position of a template-assisted NC assembly under increasing excitation fluences. The emission peak at 537 nm from the coupled NCs shows a drastic intensity increase with an increasing excitation fluence. As shown in Figure 5b, the time-integrated emission intensity of the red-shifted emission peak (wavelength range 535–570 nm) shows a linear dependence on excitation fluence, reflecting the fact that there are no excitation density-dependent nonradiative processes in the system. From the time-resolved measurements, we observed a continuous shortening of emission lifetime (Figure 5c,d top panel). The peak intensity shows a superlinear dependence on excitation fluence (Figure 5d bottom panel). These spectral and temporal dynamics, which are dependent on excitation fluence, are consistent with SF emission, as observed in spontaneously assembled ordered SLs without templates.^15^

**Figure 6:**
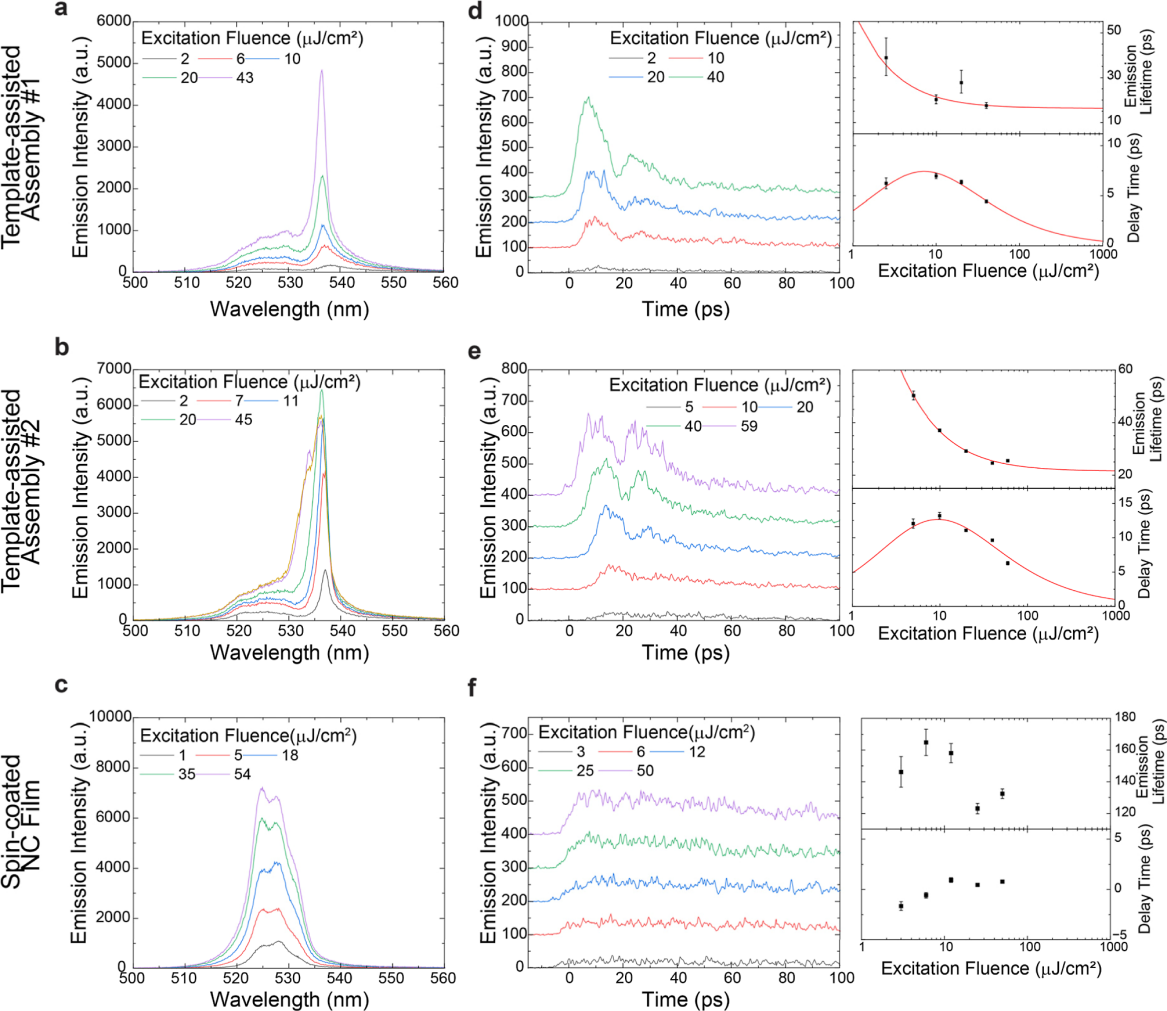
Ultrafast spectroscopy under strong femtosecond excitation at 6 K. (a,b) Time-integrated spectra of two different template-assisted NC assemblies and a spin-coated NC film (c) as a control sample. (d–f) Spectrally integrated emission time traces for different excitation fluences of both template-assisted NC assemblies (d,e) and for the spin-coated NC film (f). Each time trace is offset by additional 100 counts (d–f) in order to visually separate the curves. For both assemblies, the decay and delay times are extracted from the respective time traces and fit with an SF model function (red lines, see text). For the spin-coated film, the origin of the delay time has been set to the average value of the experimental data since the SF model function fit is not applicable.

To scrutinize the ultrafast emission dynamics of SF at even higher excitation fluence, we performed time-resolved measurements using an amplified femtosecond laser system and a streak camera instead of a pulsed diode laser with an avalanche photodiode (see Methods). Here, the excitation beam covers the whole template, precluding spatially resolved analysis. We present in Figure 6 the data from two different NC assemblies and a spin-coated control film produced with the same NC solution. As shown in Figure 6a,b, the observed red-shifted emission peak with respect to the individual NCs emission energy was similar to that for the microscopic optical characterization (Figures 4b and 5a). In contrast, the emission spectra of a spin-coated NC film (Figure 6c) and a NC assembly by the drying-mediated method (Figure S8c) mainly consist of two emission peaks with energy separation of 14–26 meV, comparable with the reported values of energy separation of excitons and trions.^35,36^ In addition, for the higher excitation fluences, we observed narrow emission peaks between 530 and 540 nm in the emission spectra from the drying-mediated NC assembly. We attribute these regularly spaced emission peaks to optical cavity modes supported by multiple internal reflections at the facets of the NC assembly due to the high refractive index contrast between the NCs and the surrounding. We have not observed periodic emission peaks similar to those of the template-assisted NC assemblies. We speculate that the difference between the spectral shapes of template-assisted NC assemblies and drying-mediated NC assemblies is caused by the cleaning process (Figure 1a(vi)). As shown in Figure S8, drying-mediated NC assemblies also show a pronounced red-shifted emission peak at 540 nm after the cleaning polymer solution is applied. Even though the SLs inside the templates do not appear damaged in optical images after removal of the residual NCs outside the templates (Figure 1c bottom), the cleaning polymer also might have an effect on the SLs inside the template, and we suspect that it could lead to a similarity of the spectra.

In terms of the temporal emission dynamics (Figure 6d,e), we observed the key fingerprints of SF, namely, accelerated decay (proportional to 1/*N*) with increasing number of excited emitters *N*, i.e., increasing excitation intensity, decreasing delay time (proportional to ln(*N*)/*N*), and emission pulse ringing, so-called Burnham Chiao ringing.^37^ Conversely, such acceleration of decay, reduction of delay, and pronounced pulse ringing effect were not observed in spin-coated NC films (Figure 6f). The experimental observations from template-assisted NC assemblies are typical signatures of SF, and therefore, we infer that SF domains are formed within the template-assisted NC assemblies.

## Conclusions

In conclusion, we developed a methodology to control the size and position of NC assemblies with prefabricated hollow, transparent template structures. We established a combination of ligands, solvent, and template structures to reliably obtain CsPbBr_3_ NC assemblies with the template-assisted method. From spectroscopic measurements at cryogenic temperatures, we observed distinct emission dynamics like lifetime shortening, superlinear increase of time-resolved emission peak intensity, and ringing by increasing the excitation fluence, all typical signatures of SF. Our results open the path toward the controlled integration of superfluorescent NC assemblies into photonics nanostructures, such as waveguides and microcavities, that will be pivotal for applications.

## Methods

### Synthesis of CsPbBr_3_ Nanocrystals

CsPbBr_3_ NCs have been synthesized according to the recently developed approach with some modifications.^29^

#### Stock Solutions

##### PbBr_2_-TOPO Stock Solution (0.04 M)

PbBr_2_ stock solution was prepared by dissolving PbBr_2_ (1 mmol, 376 mg, Sigma-Aldrich) and trioctylphosphine oxide (5 mmol, 2.15 g, TOPO 90%, Strem) in octane (5 mL, Roth) at 100 °C, followed by dilution with hexane (20 mL) and filtering through a 0.2 μL PTFE filter before use.

##### Cs-DOPA Stock Solution (0.02 M)

Cs-DOPA stock solution (0.02 M) was prepared by mixing 100 mg of Cs_2_CO_3_ (Sigma-Aldrich) together with 1 mL of diisooctylphosphinic acid (DOPA, Sigma-Aldrich) and octane (2 mL, Roth) at 100 °C followed by dilution with hexane (27 mL) and filtering through a 0.2 μL PTFE filter before use.

##### Didodecyldimethylammonium Bromide Solution (100 mg/mL, 0.215 M)

DDAB solution (100 mg/mL, 0.215 M) was prepared by dissolving 300 mg of DDAB (Sigma-Aldrich) in 3 mL of anhydrous toluene (Sigma-Aldrich).

##### Oleic Acid/Oleylamine Stock Solution

Oleic acid/oleylamine stock solution (OAc/OAm) was prepared by mixing 0.632 mL of dried oleic acid (Sigma-Aldrich) and 0.66 mL of distilled oleylamine (Strem) in 5 mL of anhydrous hexane.

##### Phosphatidyl-l-serine Stock Solution (0.05 M)

Phosphatidyl-l-serine stock solution (Ptd-l-Ser, 0.05 M) was prepared by mixing 39.6 mg of Ptd-l-Ser (Biosynth) with 1 mL of distilled mesitylene (Acros).

#### Synthesis

In a 25 mL one-neck flask, 2 mL of PbBr_2_-TOPO stock solution was combined along with 3 mL of hexane. Under vigorous stirring, 1 mL of Cs-DOPA stock solution was swiftly injected. In 2 min 30 s, a stock solution of ligands (0.15 mL DDAB in toluene or 1.2 mL OAc/OAm-solution together with 35 μL of DDAB in toluene or 0.4 mL Ptd-l-Ser in mesitylene) was added to initiate the ligand exchange on the NC surface. In 2 min 30 s after addition of the ligands, the crude solution was concentrated by evaporating hexane on a rotary evaporator down to less than 1.2 mL of residual solvent. The NCs were precipitated from the concentrated colloid by adding a nonsolvent.

#### Purification

DDAB-capped NCs were purified using acetone (crude solution/nonsolvent 1:1, v/v), followed by solubilization of the obtained NCs in cycloheptane. For OAc/OAm/DDAB-capped NCs, ethyl acetate was used (crude solution/nonsolvent, 1:1, v/v), followed by solubilization of the obtained NCs in anhydrous toluene. Ptd-l-Ser-capped NCs were purified using a mixture of ethyl acetate and acetonitrile with a volume ratio of 2:1 (crude solution/nonsolvent, 1:3, v/v), followed by solubilization of the obtained NCs in anhydrous toluene.

The concentrations of NCs were about 15 mg/mL (OAc/OAm/DDAB-capped), 10 mg/mL (DDAB-capped), and 6 mg/mL (Ptd-l-Ser). These solutions were used further for the preparation of the 3D SLs.

### Absorption and Photoluminescence Measurements

UV–vis absorption spectra were collected by using a JASCO V770 spectrometer operated in transmission mode. A Fluoromax 4 Horiba Jobin Yvon spectrofluorometer equipped with a PMT detector was used to acquire steady-state PL spectra from the solutions. The excitation wavelength was 400 nm, provided by a 150 W xenon lamp dispersed with a monochromator. Measured intensities were corrected to take into account the spectral response of the detector.

### Transmission Electron Microscopy

TEM images were collected using a JEOL JEM-2200FS microscope operated at 200 kV.

### Fabrication of 3D Hollow Template Structures

SOI substrates were treated under oxygen plasma for 5 min to obtain better adhesion of hydrogen silsesquioxane (HSQ, Dow Corning). HSQ was spin-coated onto the substrates. After e-beam exposure and development of the HSQ, the lithographically defined pattern of the HSQ was transferred to the Si device layer by inductively coupled plasma RIE with HBr. A 200 nm thick SiO_2_ layer was deposited on the substrates with ALD and e-beam evaporated deposition. The openings of the template structures were defined by EBL with CSAR (AllResist) as the resist. After the development of CSAR, the pattern was transferred to the SiO_2_ layer by RIE with CHF_3_ and Ar. Lastly, the exposed Si layer was dry-etched with XeF_2_ gas.

### Template-Assisted NC Assembly

5 μL of NC solution was drop-casted on a 10 × 10 mm substrate with template structures. The substrate was placed in a Petri dish (diameter: 90 mm; height: 20 mm) with 1 mL of toluene. The Petri dish was covered with a watch glass so that the solvent evaporated slowly. The evaporation of solvent usually takes 24 h. For the removal of excess NCs, optics cleaning polymer (Red First Contact Polymer, Photonic Cleaning Technologies) was applied on the substrate surface and peeled off after the polymer solution was dried out. This procedure cannot perfectly remove all excess NCs and NCSLs on the surface, and there some residual NCs and NCSLs remaining in some areas of the substrate surface, labeled in our measurements as “NC assemblies after applying the cleaning polymer”.

### Preparation of Spin-Coated NC Thin Films

The spin-coated NC films were prepared in a glovebox that was kept under an argon atmosphere. 20 μL of NC solution was put on a 10 × 10 mm Si substrate with a 2 μm thick thermally grown SiO_2_ layer on top. The solution was spin-coated for 60 s with a spin speed of 2000 rpm.

### STEM Cross-Section Imaging

Template structures with NCs were sliced down to lamella structures by focused ion beam (FIB), using a FEI Helios Nanolab 450S. After covering the area of interest with 120 nm soft Pt (deposited by an electron beam), a 1.5 μm thick lamella was extracted from the wafer by removing the material on both sides of the region of interest under high voltage (30 keV) and high current (9.4 nA). It was then attached to a TEM grid with Pt and thinned down first at 30 keV and 7 pA until a top view thickness of about 200 nm was reached. Finally, the lamella was gently thinned down at 5 keV and 15 pA. The lamellas were investigated with a double spherical aberration-corrected transmission electron microscope (JEOL ARM200F) operated at 200 kV. Energy-dispersive spectroscopy was carried out with a liquid-nitrogen free silicon drift detector.

### GISAXS Measurements

GISAXS patterns were acquired using a XEUSS 3.0 system (Xenocs) equipped with a Cu Kα microsource (λ = 1.54 Å) and an Eiger2 1 M detector (Si, Dectris). The beam size was set to 2.0 × 0.3 mm, with a tilting angle of 0.2° and a sample-to-detector distance of 900 mm. Data acquisition was performed over a duration of 6 h.

### Optical Image Analysis

Optical images of the template structures were taken with a digital microscope (Keyence VHX-7000). Quantitative image analysis was performed by thresholding the RGB values to discern NC from other structures and counting the unmasked pixels.

### Optical Spectroscopy

For spatially resolved PL map measurements, we used a CW diode laser with the wavelength of 405 nm as the excitation source. The excitation laser was coupled to a single-mode fiber and was focused on the sample with a 100× objective lens (Mitutoyo) to a Gaussian spot diameter with 1/*e*^2^ diameter of 2.5 μm. The sample was mounted on *XYZ* nanopositioning stages. The sample was scanned over a 15 × 15 μm area with 1 μm step size.

Time-integrated and time-resolved PL at cryogenic temperature was measured by mounting the samples in a coldfinger flow cryostat, which operates down to a temperature of 6 K. We used a fiber-coupled diode laser (PicoQuant) with a wavelength of 405 nm as the excitation source. The laser has a pulse duration of ∼50 ps, with a repetition rate of 250 kHz. The laser emission was filtered with a short-pass filter edge of 430 nm and was focused on the sample with a 100× objective lens (Mitutoyo) to a Gaussian spot with 1/*e*^2^ diameter of 4.4 μm. The emitted light from the sample was collected by the same objective and passed through a long-pass filter (Semrock) with 450 nm edge wavelength. The collected light was dispersed by a 300 lines/mm grating in a 0.75 m long monochromator, and the spectra were recorded by an EMCCD (Princeton Instruments). The PL time traces were recorded with an avalanche photodiode (MPD, time resolution of 50 ps) connected to a time-correlated single-photon counting system.

For ultrafast time-resolved measurements with higher time resolution, the samples were mounted in a helium exchange-gas cryostat, which operates at a temperature down to 6 K. As an excitation source, we used a frequency-doubled regenerative amplifier running at 400 nm with a repetition rate of 1 kHz, delivering pulses of about 100–200 fs duration. To prevent parasitic light, a short-pass filter (edge wavelength of 492 nm) was used. For both excitation and detection, we used the same focusing lens with 100 mm focal length, resulting in an excitation spot size of about 80 μm in 1/*e*^2^ diameter. The recorded PL was spectrally filtered by a long-pass filter (edge wavelength of 460 nm). For the time-resolved measurements, the emission was dispersed by a 150 lines/mm grating in a 0.3 m long monochromator and detected with a streak camera (Hamamatsu) with a nominal time resolution of 2 ps and measured Gaussian-shaped instrument response function fwhm of 4 ps. The time-integrated PL spectra were recorded by a 0.5 m long spectrograph equipped with a 300 lines/mm grating and a liquid-nitrogen-cooled CCD camera.

## Data Availability

The pre-print version of this work is: Kobiyama, E.; Urbonas, D.; Bodnarchuk, M. I.; Rainò, G.; Olziersky, A.; Caimi, D.; Sousa, M.; Mahrt, R. F.; Kovalenko, M. V.; Stöferle, T. Perovskite nanocrystal self-assemblies in 3D hollow templates. 2024, 2406.17665. arXiv. https://doi.org/10.48550/arXiv.2406.17665 (accessed Dec 12, 2024).

## Supporting Information Available

PL and absorption spectra of NCs, a TEM image of NCs, size distribution of NCs, GISAXS data, optical microscopy images, STEM images and EDS measurement results of a template-assisted NC assembly, masked optical microscope images of template-assisted NC assemblies for image analysis, detailed statistical analysis of the yield of the template-assisted assembling method, and ultrafast PL dynamics of a drying-mediated NC assembly before and after applying the cleaning polymer (PDF)
