# Supplementary material for: Perovskite Nanocrystal Self-Assemblies in 3D Hollow Templates
Source: ACS Nano. 2025 Jan 13;19(7):6748–57. doi: 10.1021/acsnano.4c07819 (PMC11867005; doi:10.1021/acsnano.4c07819)
Supplement: Supplementary file 1 — nn4c07819_si_001.pdf [file nn4c07819_si_001.pdf]

Supporting Information

Perovskite Nanocrystal Self-Assemblies in 3D Hollow Templates

Etsuki Kobiyama[1], Darius Urbonas[1], Benjamin Aymoz[2,3], Maryna I. Bodnarchuk[2,3,], Gabriele Rainò[2,3], Antonis Olziersky[1], Daniele Caimi[1], Marilyne Sousa[1], Rainer F. Mahrt[1], Maksym V. Kovalenko[2,3,*], Thilo Stöferle[1,*]*

[1] IBM Research Europe – Zurich, Säumerstrasse 4, 8803 Rüschlikon, Switzerland.

[2] Institute of Inorganic Chemistry, Department of Chemistry and Applied Bioscience, ETH Zurich, 8093 Zurich, Switzerland.

[3] Laboratory of Thin Films and Photovoltaics, Empa — Swiss Federal Laboratories for Materials Science and Technology, 8600 Dübendorf, Switzerland.

*** Corresponding Authors:** maryna.bodnarchuk@empa.ch, mvkovalenko@ethz.ch, tof@zurich.ibm.com

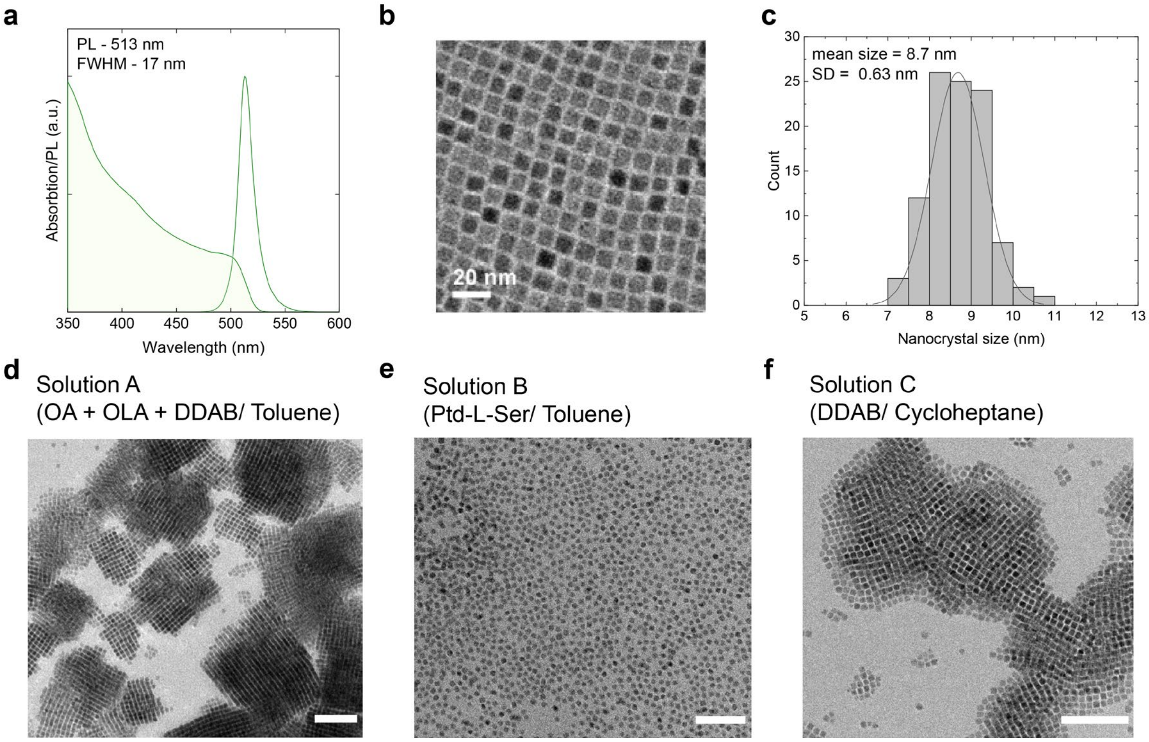

Figure S1. (a) Absorption and PL spectra of $CsPbBr_3$ NCs. (b) TEM image of $CsPbBr_3$ NCs. Scale bar: 20 nm. (c) Histogram of edge size of $CsPbBr_3$ NCs. (d-f) TEM images of drop-casted NCs from solution A (ligands: oleic acid (OA) + oleylamine (OLA) + didodecyldimethylammonium bromide (DDAB), solvent: toluene) (d), solution B (ligands: phosphatidylserine (Ptd-L-Ser), solvent: toluene) (e), and solution C (ligands: DDAB, solvent: cycloheptane) (f). The solutions were drop-casted on TEM grids. Scale bars: 100 nm.

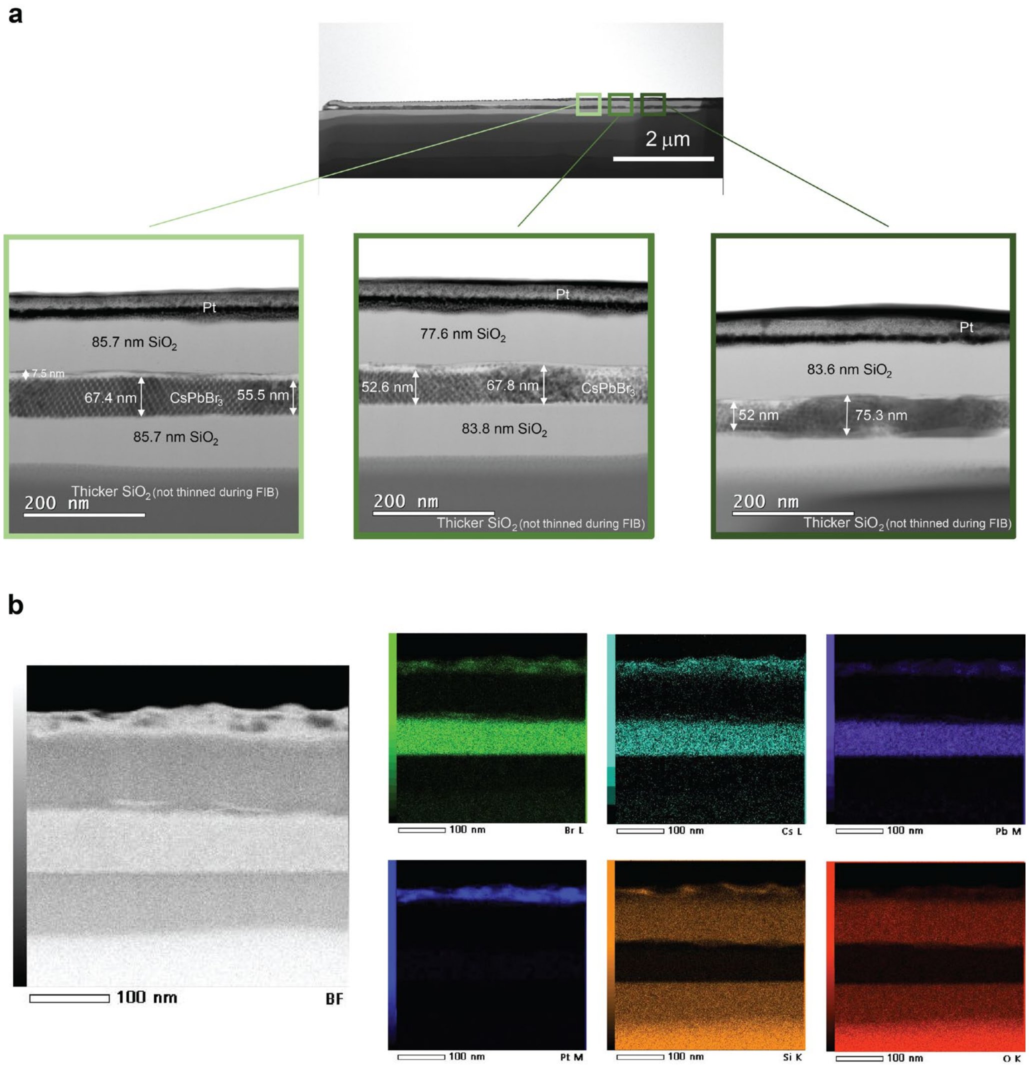

Figure S2. (a) Cross section bright field STEM images of different positions of a template-assisted NC assembly, revealing grains of various thickness. (b) Results of energy dispersive spectroscopy (EDS). The left panel shows a bright field image of the cross section of a template-assisted NC assembly. The right panels display EDS signals of different species: Br (top left, green), Cs (top center, cyan), Pb (top right, violet), Pt (bottom left, blue), Si (bottom center, orange), and O (bottom right, red).

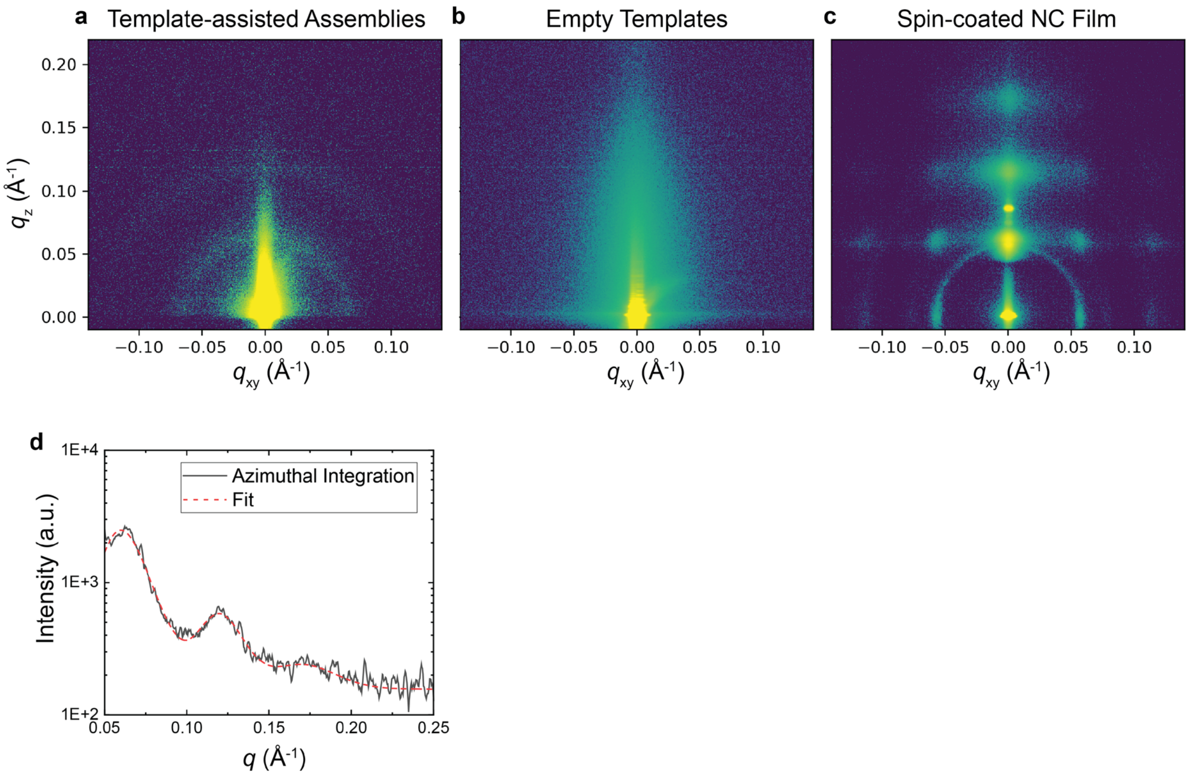

Figure S3. Grazing-incidence small-angle X-ray scattering (GISAXS). (a) GISAXS signals from template-assisted NC assemblies. (b) GISAXS signals from substrate with template structures before NC deposition. (c) GISAXS signals from a spin-coated film from the same NC solution. (d) The azimuthal integration of the GISAXS signals from template-assisted NC assemblies and 3-peak fit.

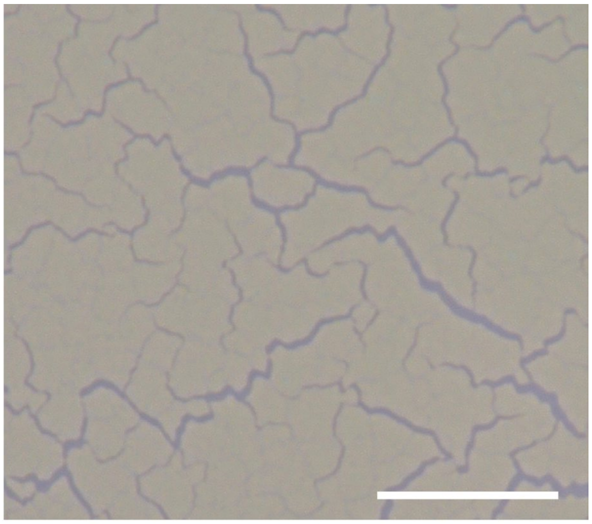

Figure S4. Optical microscope image of spin-coated NC film. Scale bar: 25 μm.

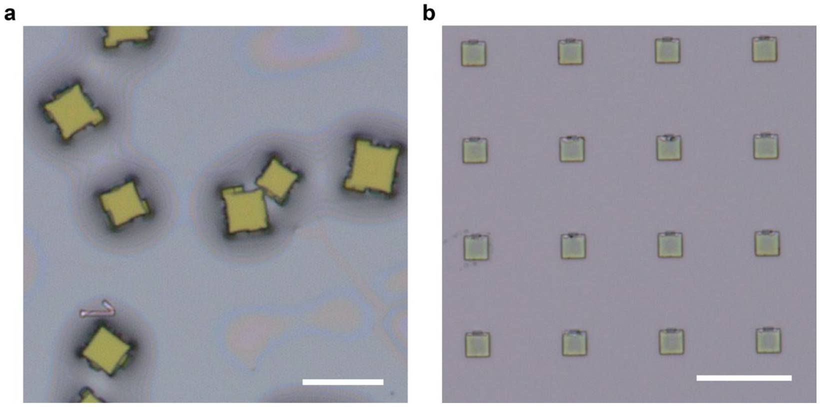

Figure S5. Optical microscope images of (a) drying-mediated NC assemblies and (b) template-assisted NC assemblies with two-times diluted solution A. Scale bar: 25 μm.

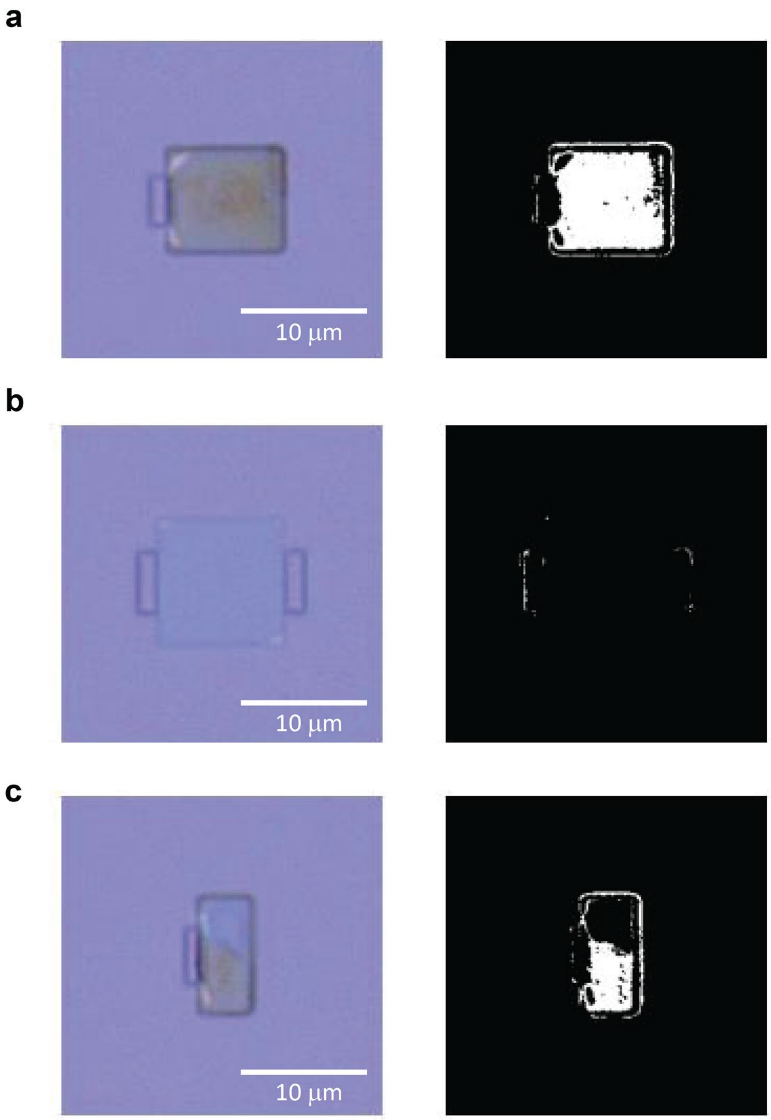

Figure S6. (a-c) Typical optical microscope image (left) and the result of binary masking the image (right) of a filled template (a), an empty template (b), and a partially filled template (c).

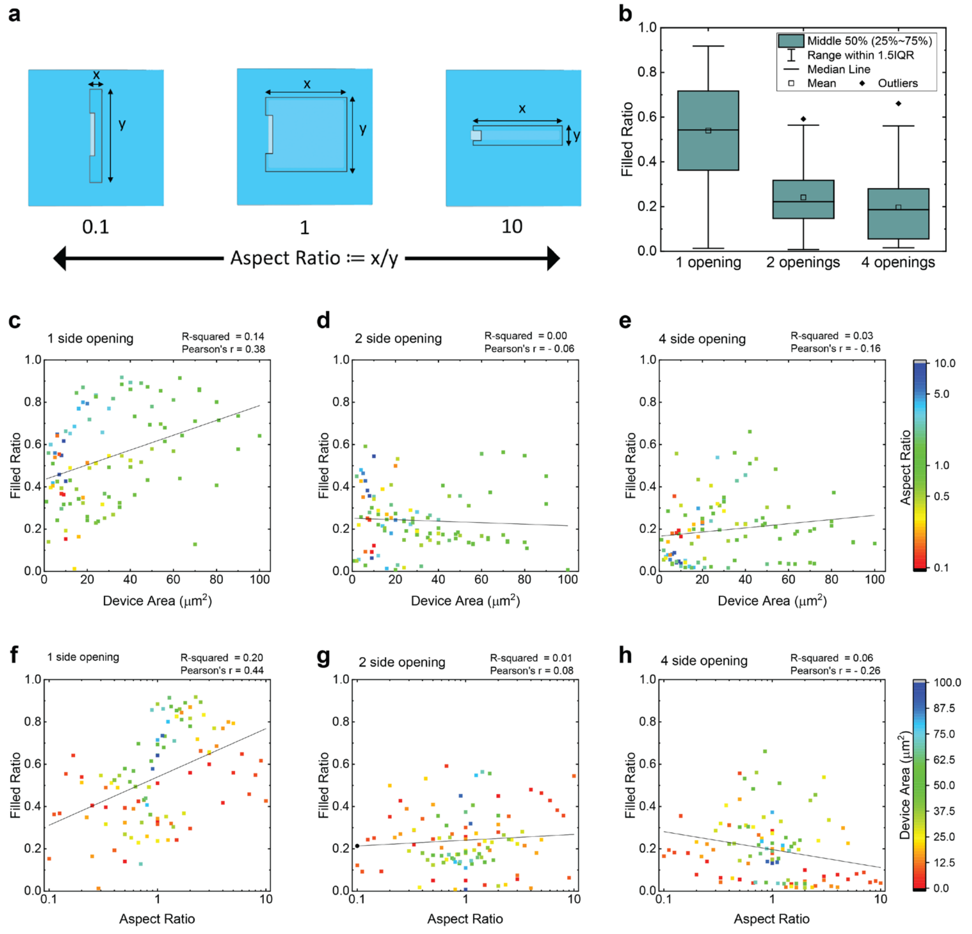

Figure S7. (a) Definition of aspect ratios of template structures. (b) Box plots summarizing the distribution of filled ratios of different design template structures. (c-e) Scatter plots of filled ratio against device area for different numbers of openings: (c) on one side, (d) on two sides, and (e) on four sides. (f-h) Scatter plots of filled ratio against aspect ratio of devices for different numbers of openings: (f) on one side, (g) on two sides, and (h) on four sides.

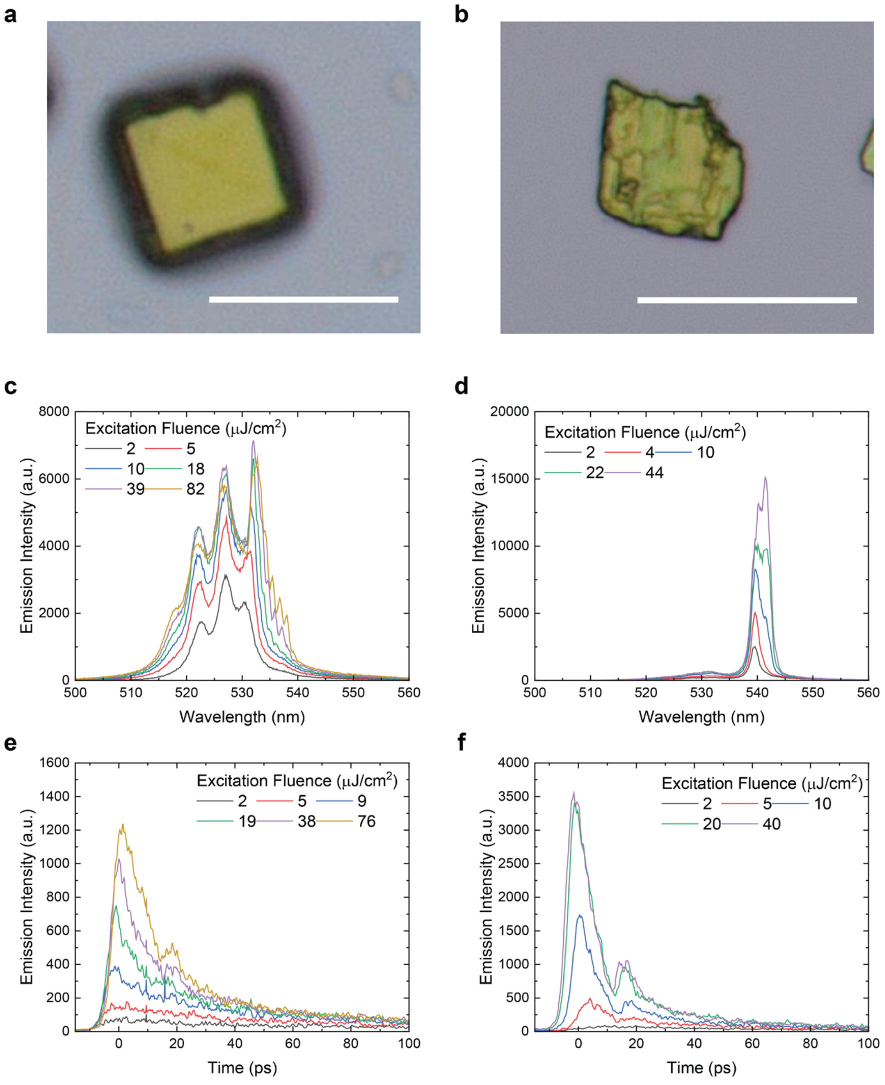

Figure S8. Ultrafast spectroscopy under strong femtosecond excitation at 6 K. (a-b) Optical microscope images of a drying-mediated NC assembly (a) and a drying-mediated NC assembly after applying the cleaning polymer (b).The scale bars are 25 μm. (c-d) Time-integrated spectra of a drying-mediated NC assembly (c) and a drying-mediated NC assembly after applying the cleaning polymer (d). (e-f) Spectrally-integrated emission time traces for different excitation fluences of a drying-mediated NC assembly (e) and a drying-mediated NC assembly after applying the cleaning polymer (f).
